# Isolation, cultivation and genomic analysis of magnetosome biomineralization genes of a new genus of South-seeking magnetotactic cocci within the *Alphaproteobacteria*

**DOI:** 10.3389/fmicb.2014.00072

**Published:** 2014-02-25

**Authors:** Viviana Morillo, Fernanda Abreu, Ana C. Araujo, Luiz G. P. de Almeida, Alex Enrich-Prast, Marcos Farina, Ana T. R. de Vasconcelos, Dennis A. Bazylinski, Ulysses Lins

**Affiliations:** ^1^Instituto de Microbiologia Paulo de Góes, Universidade Federal do Rio de JaneiroRio de Janeiro, Brazil; ^2^Laboratório Nacional de Computação Científica, Departamento de Matemática Aplicada e ComputacionalPetrópolis, Brazil; ^3^Instituto de Biologia, Universidade Federal do Rio de JaneiroRio de Janeiro, Brazil; ^4^Instituto de Ciências Biomédicas, Universidade Federal do Rio de JaneiroRio de Janeiro, Brazil; ^5^School of Life Sciences, University of Nevada at Las VegasLas Vegas, NV, USA

**Keywords:** *Magnetofaba australis* strain IT-1, magnetite, magnetosome, South-seeking magnetotactic bacteria, biomineralization genes

## Abstract

Although magnetotactic bacteria (MTB) are ubiquitous in aquatic habitats, they are still considered fastidious microorganisms with regard to growth and cultivation with only a relatively low number of axenic cultures available to date. Here, we report the first axenic culture of an MTB isolated in the Southern Hemisphere (Itaipu Lagoon in Rio de Janeiro, Brazil). Cells of this new isolate are coccoid to ovoid in morphology and grow microaerophilically in semi-solid medium containing an oxygen concentration ([O_2_]) gradient either under chemoorganoheterotrophic or chemolithoautotrophic conditions. Each cell contains a single chain of approximately 10 elongated cuboctahedral magnetite (Fe_3_O_4_) magnetosomes. Phylogenetic analysis based on the 16S rRNA gene sequence shows that the coccoid MTB isolated in this study represents a new genus in the *Alphaproteobacteria*; the name *Magnetofaba australis* strain IT-1 is proposed. Preliminary genomic data obtained by pyrosequencing shows that *M. australis* strain IT-1 contains a genomic region with genes involved in biomineralization similar to those found in the most closely related magnetotactic cocci *Magnetococcus marinus* strain MC-1. However, organization of the magnetosome genes differs from *M. marinus*.

## Introduction

Magnetotactic bacteria (MTB) are a morphologically, metabolically, and phylogenetically diverse group of prokaryotes that share the ability to synthesize intracellular, nano-sized magnetic particles called magnetosomes. Each magnetosome consists of a magnetite (Fe_3_O_4_) or greigite (Fe_3_S_4_) crystal enveloped by a lipid-bilayer membrane derived from the cytoplasmic membrane (Bazylinski and Frankel, 2004). Magnetosomes are generally organized in linear chains and orient the cell body along geomagnetic field lines while flagella actively propel the cells, resulting in so-called magnetotaxis (Bazylinski and Frankel, 2004; Schüler, 2008). MTB from the Southern Hemisphere swim antiparallel to the vertical component of the geomagnetic field toward the South and are termed South-seeking MTB (SS-MTB). In contrast, MTB from the Northern Hemisphere swim parallel to the vertical component of the geomagnetic field lines and are predominantly North-seeking (NS-MTB) (Blakemore et al., 1980). The inclination of the geomagnetic field lines is believed to direct cells downwards away from toxic concentrations of oxygen in surface waters, thereby helping them locate and maintain an optimal position in vertical gradients which is usually at or near the oxic-anoxic interface (OAI) (Blakemore, 1982; Frankel and Bazylinski, 1994; Bazylinski and Frankel, 2004). However, there are reports of SS-MTB and NS-MTB in both hemispheres (Simmons et al., 2006).

MTB are considered fastidious microorganisms (Schüler, 2008), although there has recently been a considerable increase in available cultures, including the first cultivation of a greigite producer (Lefèvre et al., 2011). The recent availability of MTB cultures has contributed to a better characterization of the physiology and biochemistry of these microorganisms. It has also contributed to an improved understanding of the evolution of MTB and of the biomineralization processes involved since differences in the sequences of magnetosome biomineralization genes in different MTB, particularly the *mam* genes, revealed a strong correlation between these magnetotaxis-related genes and phylogeny based on the 16S rRNA gene (Lefèvre et al., 2013a). Studies of magnetosome biomineralization genes in uncultivated MTB require unique approaches (Abreu et al., 2011; Jogler et al., 2011) that do not usually reveal the complete organization of biomineralization genes or genes involved in magnetotactic behavior unless the entire genome is sequenced. Moreover, because not all the magnetosome-related genes may be recognized, a direct correlation with phylogeny based on 16S rRNA gene sequences cannot be made with total accuracy.

The most characterized cultivated MTB strains are phylogenetically affiliated with the *Alphaproteobacteria* and include *Magnetococcus marinus* strain MC-1 (Bazylinski et al., 2013a), *Magnetovibrio blakemorei* strain MV-1 (Bazylinski et al., 2013b), the magneto-ovoid bacterium strain MO-1 (Lefèvre et al., 2009), *Magnetospirillum magneticum* strain AMB-1, *Magnetospirillum gryphiswaldense* strain MSR-1, *Magnetospirillum magnetotacticum* strain MS-1, *Magnetospira thiophilla* strain MMS-1 (Williams et al., 2012) and *Magnetospira* sp. QH-2 strain 1 (Ji et al., 2014). Cultivated strains belonging to *Deltaproteobacteria* include the sulfate-reducer *Desulfovibrio magneticus* strain RS-1, (Sakaguchi et al., 2002), *Candidatus* Desulfamplus magnetomortis strain BW-1 (Lefèvre et al., 2011) and enrichment cultures of the magnetotactic multicellular prokaryotes *Candidatus* Magnetoglobus multicellularis (Abreu et al., 2013). Two cultivated strains, BW-2 and SS-5, both belonging to *Gammaproteobacteria*, have also been reported (Lefèvre et al., 2012).

The biomineralization of magnetosomes is controlled by a set of highly conserved genes in magnetite-producing MTB (Richter et al., 2007; Jogler and Schüler, 2009; Jogler et al., 2009) and, as demonstrated more recently, in greigite-producing MTB as well (Abreu et al., 2011, 2013; Lefèvre et al., 2011, 2013b). In some species, the magnetosome biomineralization genes are clustered on a genomic magnetosome island (MAI), which partially supports the hypothesis of horizontal gene transfer (HGT) between various MTB presumably leading to the wide distribution of these genes among members of different phylogenetic groups (Jogler and Schüler, 2009; Jogler et al., 2009; Abreu et al., 2011). However, certain components of typical genomic islands (transposases, t-RNA sequences, integrases), such as those observed in *M. magneticum* strain AMB-1, *M. gryphiswaldense* strain MSR-1 and *D. magneticus* RS-1, are not universally shared within the MAI of all MTB (e.g., *M. marinus*; Schübbe et al., 2009). Moreover, phylogenetic analysis based on the amino acid sequences of magnetosome proteins from MTB are congruent with the phylogenetic tree based on the 16S rRNA gene sequences of the same microorganisms (Lefèvre et al., 2013a). Therefore, the evolution and divergence of magnetosome proteins and the 16S rRNA gene occurred similarly, suggesting that magnetotaxis originated monophyletically in the *Proteobacteria* phylum (Lefèvre et al., 2013a). Additional genome sequences and culture of MTB species are necessary to understand the evolution of biomineralization in *Bacteria*. Moreover, the availability of new cultures of MTB allows a better characterization of the physiology and biochemistry of these microorganisms, enabling the correlation of these features to magnetosome formation.

Despite being the most prevalent and diverse morphotype of MTB in the environment (Spring et al., 1998; Schübbe et al., 2009), there are currently only two cultivated strains of magnetotactic cocci: *M. marinus* strain MC-1 (Bazylinski et al., 2013a) and the magneto-ovoid bacterium strain MO-1 (Lefèvre et al., 2009). The complete genome sequence of the NS-MTB *M. marinus* has been reported (Schübbe et al., 2009), but further study is required to better understand the full diversity of the magnetotactic cocci as well as the ecological function and evolution of magnetosome biomineralization in the *Alphaproteobacteria*. Here, we describe both the isolation in axenic culture and the characterization of a new magnetotactic coccus, provisionally named *Magnetofaba australis* strain IT-1 that represents a new genus. We also conducted whole genome sequencing and functional annotation of genes related to magnetosome formation to gain insight into the phylogeny, physiology and biochemistry of this SS-MTB. This strain is the first cultivated SS-MTB, and the genomic data presented here are the first report of biomineralization genes in magnetotactic cocci capable of synthesizing elongated cuboctahedral magnetosomes.

## Materials and methods

### Isolation and cultivation of *magnetofaba australis* strain IT-1.

Samples of water and sediment were collected from the Itaipu Lagoon (22°57′51.90″ S 43°2′45.41″ W), a brackish to marine coastal lagoon near Rio de Janeiro, Brazil, and stored under dim light at room temperature. MTB were magnetically concentrated using a magnetic isolation apparatus described by Lins et al. (2003). After 20 min, cells were collected in a polypropylene tube. Concentrated South-seeking MTB were magnetically purified repeatedly using the racetrack technique (Wolfe et al., 1987) and inoculated at the OAI of culture tubes. Approximately 4/5 of the tubes were filled with an autotrophic semisolid oxygen concentration gradient ([O_2_]-gradient) medium. The medium was used to isolate *M. marinus* (Frankel et al., 1997) and contained bicarbonate as the major carbon source. The medium contained 5 mL of modified Wolfe's minerals elixir, 3.75 mM NH_4_Cl, 0.2 mL of 0.2% resazurin and 2 g of Bacto-Agar diluted in 1 L of artificial seawater (ASW). The medium was autoclaved, followed by the addition of 1.5 mL of 0.5 M KHPO_4_, pH 7.1, neutral fresh L-cysteine (final concentration of 0.2 g/L) and 2.68 mL of 0.8 M NaHCO_3_, 0.5 mL of vitamin solution and 2 mL of 0.01 M ferric quinate (final concentration of 20 μM). The pH was adjusted to 7.2. Cultures were incubated at 28°C until a microaerophilic band of cells was observed at the OAI and, subsequently, the bands were inoculated into a solid heterotrophic [O_2_]-gradient medium applying the dilution-to-extinction technique and shake-tubes (Seeley et al., 1991). Briefly, a band of cells were inoculated into the solid medium before it solidified (approximately at 45°C), followed by 7 serial 10-fold dilution steps. After inoculation and agitation by inversion, each tube was put on ice to solidify the medium quickly without killing a significant number of cells. Colonies grown on shake tubes were individually transferred to semi-solid heterotrophic medium. Each colony in culture was re-inoculated in fresh medium over 10 times to ensure that a pure culture was obtained. Purity of the culture was evaluated by light and electron microscopy and sequencing of the 16S rRNA gene.

The medium chosen for growth and maintenance of *M. australis* strain IT-1 was designed for heterotrophic growth, because cells grew faster and the number of magnetosome per cell was higher than in the autotrophic medium. The heterotrophic medium contained 5 mL of modified Wolfe's minerals (Frankel et al., 1997), 3.75 mM NH_4_Cl, 0.2 ml of 0.2% resazurin, 12 mM HEPES, 12 mM sodium acetate, 3.7 mM sodium succinate and 2 g of Bacto-Agar in 1 L of ASW. The medium was autoclaved, followed by the addition of 1.5 mL of 0.5 M KHPO_4_, pH 7.1, neutral fresh L-cysteine (final concentration of 0.2 g/L), 2.68 mL of 0.8 mM NaHCO_3_ and 4.8 mM Na_2_O_3_S_2_•5H_2_O. The pH was adjusted to 7.2, and 0.5 mL of a vitamin solution (Frankel et al., 1997) and 2.5 mL of 0.01 M ferric quinate were added. Cells were inoculated at the OAI, and the cultures were incubated at 28°C for at least 15 days.

Oxygen concentrations were measured using a Unisense OX 100 oxygen microsensor, with a detection limit of 0.3 μM, coupled to a micromanipulator MM33 (Unisense, Aarhus, Denmark). Measurements were carried in duplicate tubes at 24 h intervals for 8 days in semi-solid heterotrophic medium. Calibration was done by submerging the sensor in a 0.1 M of ascorbate and 0.1 M of NaOH solution (0% O_2_ saturation) and oxygenated water (100% O_2_ saturation). The oxygen concentration profile was determined to a depth of 11 mm from the culture medium surface in 200 μm steps taking 5 s for each measurement. The O_2_ microsensor was stabilized for 2–3 h before any measurement. Data were recorded in the software SensorTrace Pro v3.0.2 (Unisense)

### Light and electron microscopy

For light microscopy imaging, drops of ASW containing magnetically-enriched MTB were placed onto coverslips and imaged with Zeiss Axioplan 2 or Zeiss Axioimager microscopes (Carl Zeiss, Göttingen, Germany), both equipped with differential interference optics. A bar magnet was used to direct MTB to the edge of the drop where they accumulated. Transmission electron microscope (TEM) imaging of cells and elemental analysis of both magnetosomes and cell inclusions were performed in unfixed and unstained samples with a Jeol 1200 EX transmission (Jeol, Peabody, MA, USA) electron microscope equipped with a Noran accessory for energy-dispersive X-ray analysis (EDS) (Thermo Scientific, Palm Beach, FL, USA). Cells were placed onto formvar-coated electron microscopy 300 mesh copper grids, rinsed with distilled water and air-dried. Observations were performed at 100 kV, and spectra were acquired using a spot size of approximately 80 nm in diameter. For magnetosome measurements, the grids were observed with a Morgagni TEM (FEI Company, Hillsboro, OR, USA) operating at 80 kV, and images were analyzed using ImageJ software (rsb.info.nih.gov/ij/). Crystal size and shape factor were calculated as (length + width)/2 and width/length, respectively. Analyses of variance were performed using Graphpad InStat version 3.0.

For energy-filtering transmission electron microscopy (EFTEM), unstained ultra-thin sections were imaged with a Zeiss EM902 (Carl Zeiss, Göttingen, Germany) TEM equipped with a mirror-prism. Iron and oxygen maps were calculated using the three-window method with iTEM-EFTEM software (Olympus Soft Imaging Solutions GmbH, Münster, Germany). For high-resolution TEM (HRTEM), ultra-thin sections obtained as described in Abreu et al. (2013) were placed on formvar-coated copper grids and imaged in a FEG-Titan 80-300 (FEI Company, Hillsboro, OR, USA) TEM operated at 300 kV. All images were digitized with a 2kx2k Gatan UltraScan 1000 CCD camera (Gatan, Pleasanton, CA, USA) using the Digital Micrograph software (Mitchell, 2008). FFT from magnification-calibrated images was obtained using the same software.

### 16S rRNA phylogenetic analysis, genome sequencing and comparative analysis of genes related to magnetosome formation

The 16S rRNA gene was amplified from *M. australis* strain IT-1 using bacterial specific primers 8bF (5′-GRGTTTGATCCTGGCTCAG-3′) and 1512uR (5′-ACGGHTACCTTGTTACGACTT-3′). PCR products were cloned into pGEM-T Easy vector (Promega Corporation, Madison, WI) and sequenced using Macrogen sequence service (Macrogen, Korea). The alignment of 16S rRNA gene was performed using CLUSTAL W with BioEdit sequence alignment editor (Hall, 1999). A phylogenetic tree was constructed using MEGA version 5.2 (Tamura et al., 2011). We used the maximum likelihood statistical method based on Kimura 2 parameters (Kimura, 1980) with Gamma distribution and invariant sites (K2 + G + I) for analyses. The bootstrap value was calculated with 1000 replicates.

For DNA preparation for pyrosequencing, *M. australis* strain IT-1 was grown in semisolid medium. After 15 days of growth, bands from different tubes were removed, concentrated by centrifugation and washed several times with sterile distilled water. DNA samples were prepared according to Chen and Kuo (1993). *M. australis* strain IT-1 DNA was sequenced on a 454 GS FLX System sequencer (Roche Diagnostics GmbH/454 Life Sciences Corporation, Branford, CT, USA). The DNA sequences were analyzed with the SABIA (System for Automated Bacterial Integrated Annotation) platform (Almeida et al., 2004). Amino acid sequences of the MAI proteins from *M. magneticum* strain AMB-1 (AP007255) (Matsunaga et al., 2005), *M. gryphiswaldense* strain MRS-1 (AM085146) (Lohβe et al., 2011), *M. magnetotacticum* strain MS-1 (NZ_AAAP01003731) (Bertani et al., 2001), *M. marinus* strain MC-1 (NC_008576) (Schübbe et al., 2009), *M. blakemorei* strain MV-1 (FP102531) (Jogler et al., 2009), Gammaproteobacteria strain SS-5 (AFX88983—AFX88992) and *M. australis* strain IT-1 were used for identity, positives and *E*-value analysis through Blastp. Other sequences used in this work include *Ca.* M. multicellularis (HQ336745 and HQ336746) (Abreu et al., 2011), *D. magneticus* strain RS-1 (AP010904) (Nakazawa et al., 2009), *Ca.* D. magnetomortis strain BW-1 (HF547348) and strains ML-1 (JX869936—JX869937) and FH-1 (KC196864—KC196902) (Lefèvre et al., 2013b). A phylogenetic tree of concatenated MamABEIKMPQ amino acid sequences was constructed using the maximum likelihood statistical method based on WAG (Whelan and Goldman, 2001) with frequencies and gamma distribution (WAG+G+F) for analyses. Bootstrap value was calculated with 1000 replicates. The sequence of the MAI region has been submitted to GenBank/NCBI under the accession number KF933436.

## Results

### Isolation, growth and phylogenetic analyses of strain IT-1

**Figure 1:**
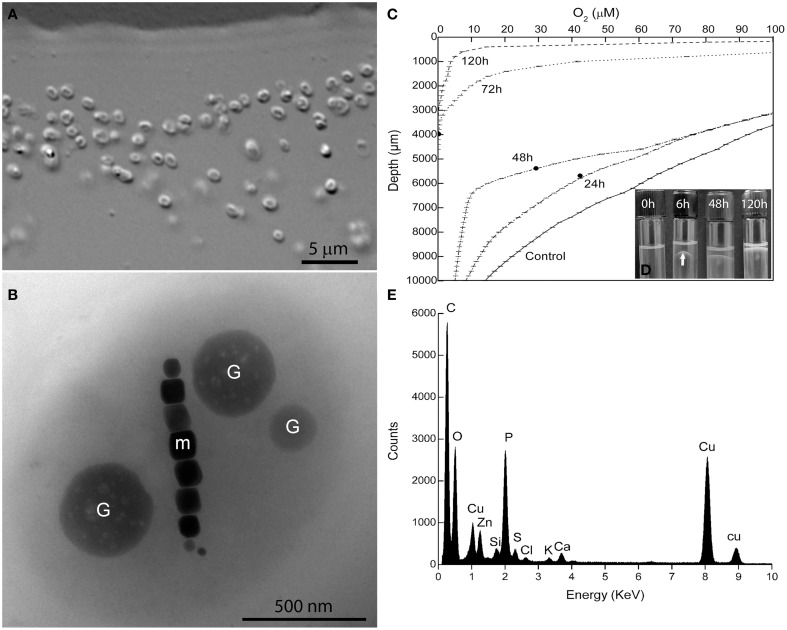
**Characterization of *Magnetofaba australis* strain IT-1**. **(A)** Differential interference contrast microscopy of a pure culture showing coccoid to ovoid cells. **(B)** Whole-mount transmission electron microscopy image of strain IT-1 showing a chain of elongated octahedral magnetosomes (m) and three conspicuous granules containing phosphorus (G). Oxygen concentration over time **(C)** and band formation **(D)** during strain IT-1 growth in semisolid heterotrophic medium. The points in the lines represent the position of the band in the culture medium at a given time. Control is represented by a non inoculated tube. Note the band with magnetotactic cells (arrow) after 6 h of inoculation. **(E)**. Energy dispersive X-ray microanalysis spectrum of the phosphorus-rich granules. Ca, Zn, and K are cations associated with the granules. Cu peaks come from the supporting grid. The silicon peak is an artifact of the Si (Li) solid state detector used to collect X-rays.

Magnetotactic cocci were the dominant MTB morphotype in the environmental samples. Occasionally, we detected magnetotactic multicellular prokaryotes, as previously described (Lins et al., 2007). After separation using the magnetic “racetrack” (Wolfe et al., 1987), magnetically-enriched cocci were inoculated at the OAI of the semisolid autotrophic medium. Four weeks later, microaerophilic bands of coccoid MTB were observed and were then inoculated in semisolid heterotrophic [O_2_] gradient medium in which the culture was maintained. Cells formed individual colonies in shake tubes of heterotrophic medium (see experimental procedures for details). Single colonies were re-inoculated in fresh semisolid medium and resulted in pure cultures of a magnetotactic coccus with an average size of 1.4 ± 0.3 × 1.1 ± 0.3 μm (*n* = 130) as observed by light microscopy (Figure 1A) and confirmed by TEM (Figure 1B). The morphology of cells observed by TEM resembles a “faba” bean, showing well-defined convex and concave surfaces (Figure 1B). Cells contain intracellular granules (Figure 1B) filled with phosphorus as detected by EDS (Figure 1E).

**Figure 2:**
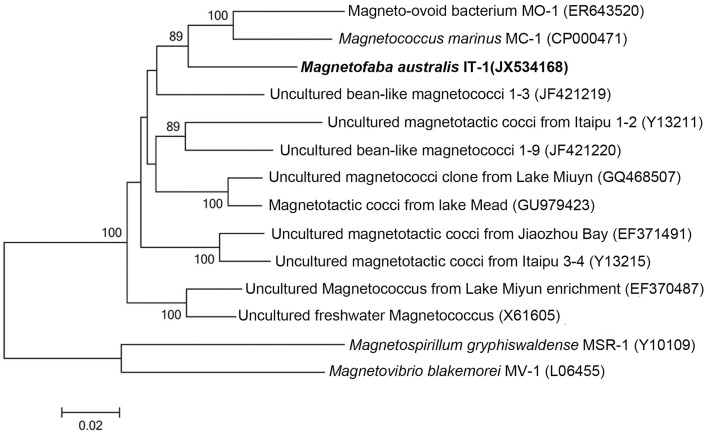
**Phylogenetic analysis based on the 16S rRNA gene of strain IT-1 that forms a new genus in the *Alphaproteobacteria***. Bootstrap values at nodes are percentages of 1000 replicates; values higher than 70 are shown at the nodes. GenBank accession numbers are given in parentheses. The phylogenetic tree was constructed using the maximum likelihood method algorithm. The scale bar indicates 0.02 substitutions per nucleotide position.

The 16S rRNA gene of the culture was amplified, cloned, and sequenced for phylogenetic analyses. Approximately 50 clones were sequenced. These sequences were 99% similar, confirming the culture was pure. A consensus sequence was generated (accession number: JX534168) and phylogenetic analysis showed that strain IT-1 is phylogenetically affiliated with the *Alphaproteobacteria* (Figure 2). The 16S rRNA gene sequence of strain IT-1 is 93% similar to the sequence of an uncultured magnetotactic coccus collected from intertidal sediments of the Yellow Sea in China (Zhang et al., 2012; accession number JF421219) and 92% similar to sequences of the cultured species *M. marinus* strain MC-1 and MO-1 (accession numbers CP000471 and EF6435202, respectively). Thus, strain IT-1 represents a new genus of the magnetotactic cocci (and MTB in general). The name *Magnetofaba australis* gen. nov., sp. nov., is proposed for strain IT-1 (Ma. gne. to. faba Gr. n. magnês -êtos, a magnet; N.L. pref. magneto-, pertaining to a magnet; N.L. fem. N. faba, a faba bean; aus.tra'lis. L. masc. australis of Southern or of the south, which refers to the polar south-seeking magnetotaxis behavior and because the bacterium was isolated from South hemisphere).

*Magnetofaba australis* strain IT-1 grows as a microaerophilic band of cells in semisolid medium (Figure 1D). It grows slowly chemolithoautotrophically, using thiosulfate as electron donor and sodium bicarbonate as the major carbon source, forming a fine band of cells at the OAI at least 4 weeks after incubation. Under these conditions, cells biomineralize 6 ± 4 magnetosomes/cell (*n* = 100). Heterotrophic growth was also observed using sodium acetate and sodium succinate as the carbon source; cells grown under these conditions produced 9 ± 4 and 7 ± 3 magnetosomes per cell, respectively (*n* = 100 for both). When cells were grown in heterotrophic medium containing both sodium acetate and succinate, a band of magnetotactic cells, which contained 10 ± 3 magnetosomes/cell (*n* = 100), was observed at the OAI after 24 h. This band gradually moved toward the surface of the culture medium after 8 days of incubation (Figure 1D).

The oxygen concentration in the band was measured over 8 days (Figure 1C) in the heterotrophic medium; cells were initially inoculated at the OAI, in which [O_2_] was less than 3 μM. During the first 6 h after inoculation, cells moved up approximately 3 mm, forming a “bell-shaped” band (Figure 1D) in the medium ([O_2_] = 50 ± 5 μM). After 24 h, the band was positioned between 24.6 ± 0.7 and 43 ± 1 μM O_2_, with a less bent bell-shape. 48 h later, the band was located in 29.2 ± 2.7 μM of [O_2_]. After 72 h, the bell-shaped band became a flat band positioned at [O_2_] between 9.4 ± 1.5 μM. Until this time, the band did not reach the meniscus of the culture medium. As the cells grew (up to 168 h), O_2_ was consumed, and the dense population of cells reached the meniscus, presumably to use oxygen present in the headspace of the tube (Figure 1D). At 72 h of incubation the cells of *M. australis* have consumed near 90% of oxygen ([O_2_] < 9.4 ± 1.5 μM), and the band appears thicker than in 24 and 48 h. With 72 h, it is likely that the magnetite production also increased, given the higher number of cells and that the population remained responding to the magnetic field at the end of the experiment. Therefore, *M. australis* strain IT-1 can grow and synthetize magnetite with [O_2_] below 10 μM, similar to the *Magnetospirillum* species, which requires microaerobic conditions (2–7 μM O_2_) to grow and synthesize magnetite (Schüler and Baeuerlein, 1998).

In hanging drop assays under oxic conditions, *M. australis* strain IT-1 exhibited South-seeking polar magnetotaxis swimming under the magnetic field of a bar magnet with a fast back and forth swimming pattern near the edge of the drop. *M. australis* swims at average speeds of 186 μm.s^−1^ ± 63 (*n* = 50) and can reach 300 μm.s^−1^. Cells are propelled by two bundles of lophotrichous flagella, each at one extremity of the cell. A helical trajectory was observed when movement was recorded with a CCD camera using dark-field microscopy.

### Magnetosomes

**Figure 3:**
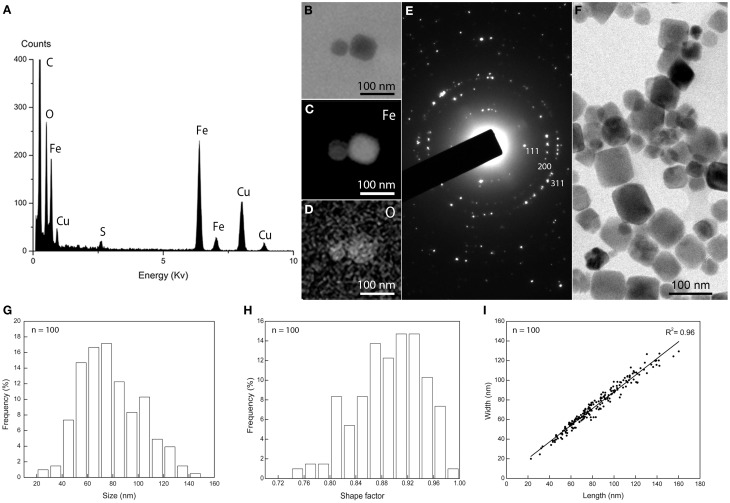
**Magnetosome biomineralization in *Magnetofaba australis* strain IT-1**. **(A)** Energy dispersive X-ray spectrum showing Fe and O as the main elements in the magnetosomes. Cu originates from the grid bar. **(B)** Elemental mapping by EFTEM of a magnetosome showing the distribution of iron **(C)** and oxygen **(D)**. **(E)** Electron diffraction pattern of isolated magnetosomes shown in **(F)**. **(G)** Size distribution, **(H)** shape factor distribution, and **(I)** scatter plot of length and width of magnetosomes in *Magnetofaba australis* strain IT-1 grown in heterotrophic medium.

**Figure 4:**
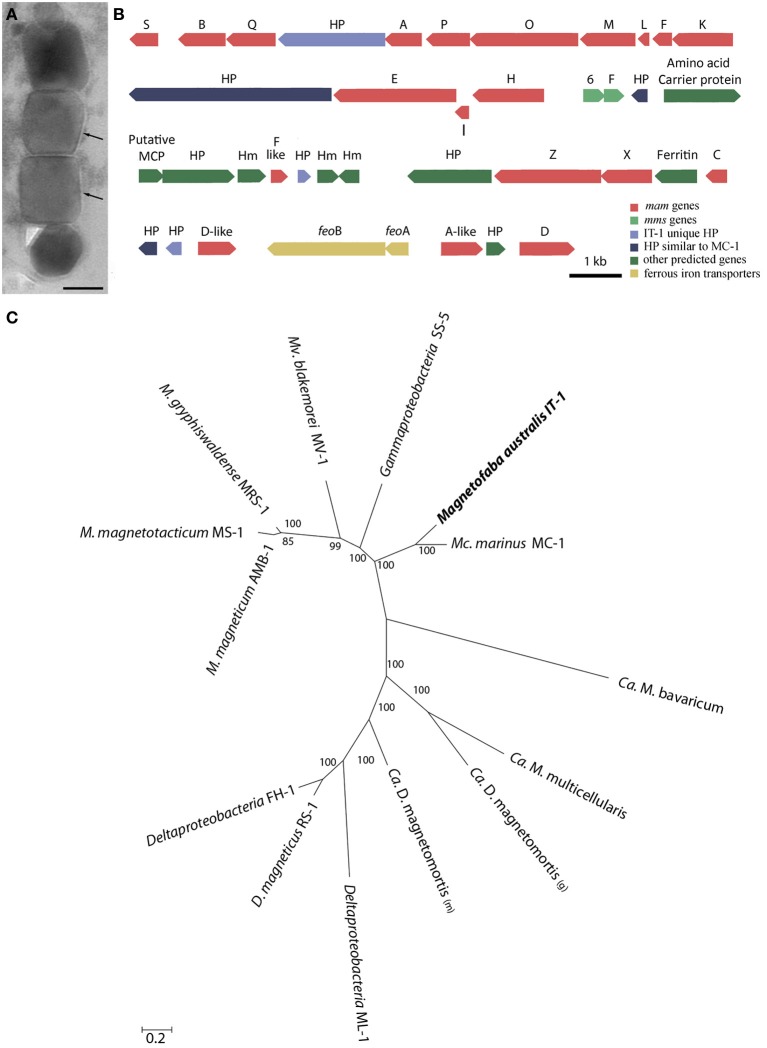
**Mam genes in *Magnetofaba australis* strain IT-1**. **(A)** Ultra-thin section image of a magnetosome chain showing the membrane (arrow) surrounding each particle. **(B)** Organization of open reading frames (ORFs) containing putative magnetosome-related genes. HP, hypothetical proteins. **(C)** Maximum likelihood phylogenetic analysis based on concatenated amino acid sequences encoded by conserved MamABEIKMPQ genes using the statistic method WAG+G+F. Note that *M. australis* strain IT-1 and *M. marinus* strain MC-1 share a common ancestor, indicating a similar biomineralization process. Bootstrap values at nodes are percentages of 1000 replicates. The scale bar indicates 0.2 substitutions per amino acid position.

Cells of *M. australis* strain IT-1 each produce a single chain of magnetosomes (see Figure 1B). Each chain consists of 10 ± 3 magnetosomes (*n* = 100) in cells grown heterotrophically in semi-solid [O_2_] gradient medium. Energy-dispersive X-ray analysis (Figure 3A) and elemental mapping by EFTEM (Figure 3B) confirmed that the magnetosomes contain iron (Figure 3C) and oxygen (Figure 3D). Electron diffraction (Figure 3E) of isolated magnetosome crystals (Figure 3F) were indexed based on standard cubic system for magnetite. Distances and angles between spots were consistent with magnetite (Fe_3_O_4_). Approximately 4% defective twins and multiple twin magnetosomes are observed in *M. australis* strain IT-1. The crystals are octahedral particles elongated along the <111> axis. Figure 3G shows the size distribution of magnetosomes (*n* = 100), estimated by calculating the best fit of an ellipse (major axis = length; minor axis = width). The average size [(length + width)/2] was 78 ± 24 nm (average length = 83 ± 26 nm; average width 74 ± 23 nm). Figure 3H shows the shape factor distribution (average of width/length = 0.89 ± 0.05), and Figure 3I shows the scatter plot of length and width (adjustment *r*^2^ = 0.962). Magnetosomes in *M. australis* strain IT-1 are each enveloped by a membrane, as shown in TEM images of ultra-thin sections (Figure 4A).

### Magnetosome genes

Comparative genome analysis of *M. australis* strain IT-1 with other magnetotactic *Alphaproteobacteria* based on genes related to magnetotaxis and magnetosome synthesis revealed a genomic region of 40.399 Kb that contained both genes associated with magnetosome biomineralization as well as those that encode some hypothetical proteins present in the putative MAI of *M. marinus*. This region contains 39 genes, 22 of which show a high degree of similarity with the biomineralization-related genes of magnetotactic *Alphaproteobacteria (mam* genes and *mms* genes; Table 1). This region also contains eight hypothetical proteins, four with high similarity values to hypothetical proteins in the putative MAI of *M. marinus* and four that are not found in any known MTB. A *feo*AB gene cluster was also identified in this region, similar to that found in *Magnetospira* sp QH-2, *Ca.* Mg. multicellularis and *Ca.* D. magnetomortis. Figure 4B shows the organization of the genes in *M. australis* strain IT-1. The *mam*AB-like gene cluster has the same gene organization found in *M. marinus* (*mamK, mamF, mamL, mamM, mamO, mamP, mamA*, HP, *mamQ, mamB and mamS*) (Schübbe et al., 2009), except that *mamT* is absent. The *mamC* gene is located in the *mam*CXZ gene cluster, similar to *M. blakemorei* (Jogler et al., 2009), and not in the *mam*HIEC gene cluster as in *M. marinus*. The *mms*6 gene cluster and the 10 genes encoding MamF-like protein, chemotaxis protein, signal transduction proteins and three hypothetical proteins are located between the *mam*AB and *mam*CXZ gene clusters. At the end of the *mam*CXZ gene cluster, genes encoding MamD-like, FeoB, FeoA, MamA-like and MamD proteins are present. All predicted proteins related to biomineralization and magnetotaxis genes described here for *M. australis* strain IT-1 share the highest similarity to those of *M. marinus*, including MamA-like, MamD-like, MamF-like, FeoB and FeoA proteins. A notable exception is MamC, which is more related to that from *M. magneticum* strain AMB-1 (coverage 86%, identity 63%, *E*-value 5e-038).

**Table 1:** **Comparative analysis of biomineralization genes found in *Magnetofava australis* strain IT-1 and other cultivated magnetotactic bacteria with their genomes sequenced**.

**IT-1**	**MC-1**			**MV-1**			**AMB-1**			**MS-1**			**MSR-1**			**SS-5**		
**Protein (locus number)**	***E*-value**	**Coverage (%)**	**Identity (%)**	***E*-value**	**Coverage (%)**	**Identity (%)**	***E*-value**	**Coverage (%)**	**Identity (%)**	***E*-value**	**Coverage (%)**	**Identity (%)**	***E*-value**	**Coverage (%)**	**Identity (%)**	***E*-value**	**Coverage (%)**	**Identity (%)**
MamS (2777)	**1E-061**	**90**	**58**	1E-035	65	44	1E-023	64	40	5E-024	64	40	2E-023	62	38	–	–	–
MamB (5564)	**6E-169**	**98**	**73**	2E-112	98	52	5E-100	97	46	2E-063	92	38	3E-101	100	45	2E-128	98	59
MamQ (2780)	**6E-128**	**96**	**62**	2E-053	91	34	7E-048	84	34	2E-043	67	37	2E-050	91	33	6E-053	67	44
MamA (2782)	**6E-089**	**98**	**58**	2E-048	98	35	2E-054	95	39	6E-060	97	38	1E-054	96	39	1E-031	96	29
MamP (5510)	**3E-123**	**98**	**64**	4E-062	67	52	6E-047	67	43	1E-047	67	43	9E-048	67	42	2E-060	76	46
MamO (5499)	**0.0**	**99**	**57**	1E-033	42	27	6E-121	97	35	4E-121	97	35	2E-115	97	35	-	-	-
MamM (5508)	**8E-170**	**95**	**69**	2E-110	96	47	2E-101	89	48	1E-090	83	47	2E-102	84	50	4E-127	86	59
HP Similar to MamL (2787)	**5E-031**	**93**	**74**	7E-016	96	40	6E-007	95	28	1E-006	95	28	7E-006	98	32	–	–	–
MamF (2788)	**6E-045**	**80**	**70**	–	–	–	3E-029	96	46	9E-028	74	52	5E-029	83	48	–	–	–
MamK (2789)	**0.0**	**100**	**82**	5E-113	97	46	7E-132	99	51	1E-122	92	51	6E-129	99	50	5E-121 (K1)	96	52
MamE (2791)	**0.0**	**100**	**48**	4E-092	98	32	1E-095	98	35	6E-096	98	35	1E-066	90	41	6E-064	97	50
MamI (2792)	**9E-029**	**66**	**81**	2E-025	78	62	4E-014	82	51	4E-014	82	51	2E-014	71	58	2E-013	63	65
MamH (5506)	**0.0**	**100**	**70**	4E-160	97	53	3E-157	95	55	1E-129	81	54	1E-155	96	54	–	–	–
HP similar to Mms6 (2798)	**9E-011**	**28**	**61**	4E-004	26	40	1E-006	36	56	8E-007	36	56	0.12	36	52	–	–	–
MmsF (2799)	**7E-056**	**83**	**76**	5E-039	81	52	1E-036 0957	79	52	8E-035	79	53	2E-035	85	52	–	–	–
MamF-like (2808)	**6E-032**	**77**	**47**	3E-026 MmsF-like	96	39	2E-025 MamF	91	45	7E-026 MamF	91	44	1E-022 MmsF	83	40	–	–	–
MamZ (2814)	**0.0**	**95**	**61**	1E-171	94	42	0.0	93	51	0.0	93	51	0.0	93	51	–	–	–
MamX (2815)	**6E-104**	**100**	**48**	3E-025	54	45	2E-056	99	35	2E-010	31	29	1E-057	99	34	–	–	–
MamC (2817)	4E-028	93	62	1E-015	82	62	**5E-032**	**86**	**63**	6E-032	82	63	4E-034	87	62	–	–	–
MamD-like (2822)	**1E-018**	**97**	**42**	–	–	–	–	–	–	–	–	–	–	–	–	–	–	–
FeoB (2827)	**81-144**	**99**	**59**	6E-112	97	50	5E-087	98	45	3E-090	97	45	1E-087	98	45	2E-092	98	44
FeoA (2828)	**3E-030**	**55**	**61**	7E-015	61	40	4E-018	58	40	1E-012	52	39	2E-012	60	40	–	–	–
MamA-like (2830)	**9E-041**	**65**	**47**	1E-013 MamA	57	30	1E-020 MamA	66	31	1E-020 MamA	66	31	1E-020 MamA	66	31	6E-013	56	30
MamD (2833)	**4E-038**	**92**	**38**	3E-014	96	39	6E-029	63	35	3E-029	63	35	1E-028	68	35	–	–	–

Bold values are related to most similar proteins between M. australis strain IT-1 and other MTB according to blastp.

The coverage, identity and *E*-value of the Blastp analysis of predicted Mam proteins from magnetotactic *Alphaproteobacteria* and the *Gammaproteobacteria* strain SS-5 were analyzed (Table 1). *M. australis* MamA-like, MamD-like and MamF-like proteins were compared to MamA-like, MamD-like and MamF-like of *M. marinus*, and MamA, MamD and MamF proteins of other MTB. The *M. australis* MamA-like predicted protein is closely related to the *M. marinus* protein sequence (coverage 65%, identity 47%, *E*-value 9e-041), while MamD-like is only related to the MamD-like from *M. marinus* (coverage 97%, identity 42%, *E*-value 1e-018). For some MTB, *M. australis* MamF-like sequences were more similar to MmsF or MmsF-like proteins (e.g., from *M. gryphiswaldense* and *M. blakemorei*). MamF and MmsF of *M. gryphiswaldense* MSR-1 have already been reported to share 65% identity (Murat et al., 2012). *M. australis* strain IT-1 MamA-like predicted proteins with MamA proteins share 30% identity (coverage 57%, *E*-value 7E-018), while MamD-like predicted proteins share 73% identity with MamD (coverage 23%, *E*-value 7E-05), and MamF-like predicted proteins share 33% identity (coverage 89%, *E*-value 2E-016) and 39% identity (coverage 90%, *E*-value 1E-021) with MamF and MmsF, respectively.

Phylogenetic analysis based on concatenated conserved Mam proteins (MamABEIKMPQ) of other MTB showed that *M. australis* strain IT-1 clusters with other *Alphaproteobacteria* that produce magnetite and is most related to *M. marinus* strain MC-1. The *Gammaproteobacteria* strain SS-5, which synthesizes cuboctahedral magnetite magnetosomes, groups with the *Alphaproteobacteria.* Interestingly, after *M. marinus*, MamB, MamQ and MamM of *M. australis* strain IT-1 have the most similarity with MamB of strain SS-5 (coverage 98%, *E*-value 2E-128, identity 59%), MamQ (coverage 67%, *E*-value 6E-053, identity 44%) and MamM (coverage 86%, *E*-value 4E-127, identity 59%). The phylogenetic tree (Figure 4C) with concatenated conserved *mam* genes does not show a clear evolutionary event that divides magnetotactic strains producing cuboctahedral and prismatic hexagonal crystals because bacteria such as *M. blakemorei* and *M. marinus* do not form a separate branch. The evolutionary relationship between *M. australis* and *M. marinus* suggests a recent divergence between the cellular magnetosome biomineralization machinery in these species.

## Discussion

The number of MTB isolated in culture has recently increased (from 1978 to 2009, 11 MTB were available in axenic cultures; in 2012 this number was 25; Lefèvre and Long-Fei, 2013). However, all cultured MTB were isolated in the Northern Hemisphere and originally showed NS magnetotaxis. This work presents the first isolation of a SS-MTB from the Southern Hemisphere. The new isolate is phylogenetically affiliated with the *Alphaproteobacteria* class of the *Proteobacteria* phylum, a division that contains almost all known Fe_3_O_4_-producing MTB (DeLong et al., 1993; Spring et al., 1998), and clearly represents a new genus based on 16S rRNA gene sequence similarities. This new coccus represents a third phylogenetic group of MTB occurring in the Itaipu Lagoon (Spring et al., 1998). *M. australis* strain IT-1 is distinct from all the other cultivated magnetotactic cocci examined to date because of its South-seeking polar magnetotactic behavior, it has “faba bean” cell morphology and elongated cuboctahedral magnetite magnetosomes. Based on its 16S rRNA gene sequence, *M. australis* is more related to an uncultured magnetotactic coccus found in the intertidal sediments of the Yellow Sea in China (93% similarity; Zhang et al., 2012). This uncultured bacterium also shows a bean-like morphology and produces magnetite magnetosomes (Zhang et al., 2012). However, magnetosome crystal morphology, size, shape factor, magnetosome number and swimming speed in *M. australis* are different from the coccus described by Zhang et al. (2012). The close phylogenetic relationships may not be significantly associated to the biomineralization genes, which may result in variations in the regulation of crystal morphology between these MTB. Hopefully, physiological studies and genomic analysis of these MTB will result in information that advances the understanding of biomineralization in bean-like magnetotactic cocci.

*Magnetofaba australis* strain IT-1 has a swimming speed similar to that observed in strain MO-1 (Lefèvre et al., 2009), higher than speeds found in other magnetotactic cocci (Zhang et al., 2012). Possibly, a highly coordinated flagella rotation is necessary to allow this high swimming speed. The high swimming speed would be advantageous for the survival of *M. australis* strain IT-1 because it would enable the cell to escape quickly from unfavorable environment conditions. Most cells of *M. australis* strain IT-1 (over 80%) has a South-seeking behavior when observed in hanging drop assays under oxic conditions, but we have also found North-seeking cells in the culture flasks. Further studies are necessary to compare the swimming behavior and orientation of magnetotactic cocci, along with their flagellar apparatus at the genetic and structural levels. We believe that such studies can now be performed because of the available SS-MTB cultures.

The role of biomineralization and magnetotaxis genes in MTB is not only key in the determination of how magnetosomes are formed in MTB but also important in understanding the evolution of magnetotaxis (Lefèvre and Bazylinski, 2013). Although several recent reports have addressed this issue (Lefèvre et al., 2013a,b), only a relatively small number of MTB species have been considered thus far. However, advances in the culturing of new strains promises to improve the low number of species available for evolutionary studies. *M. australis* strain IT-1 is the first MTB isolated in axenic culture that produces cuboctahedral magnetite magnetosomes whose magnetosome biomineralization genes have been sequenced. New data on the magnetosome biomineralization genes of coccoid or ovoid MTB increases our understanding of the biomineralization processes in MTB in general. For example, *M. marinus* and *M. australis* share several hypothetical proteins, not found in other MTB that may have key functions in biomineralization or magnetotaxis like the hypothetical protein between MamE and MamK (locus 02790), the hypothetical protein between MmsF and the Amino acid carrier protein (locus 02801), the Amino acid carrier protein (locus 02803), a hemerythrin-like (locus 02811), and a ferritin-like (locus 02816).

The analysis of the putative functions of *mam* genes is also important in the interpretation of the evolution of magnetotaxis. Variations in both the order and sequence of *mam* genes between *M. australis* and the closely related *M. marinus* could explain differences between magnetosome crystal morphology in the two species. The MamC predicted protein sequence of *M. australis* is more similar to that of *M. magneticum* strain AMB-1, which is particularly interesting because cultivated *Magnetospirillum* species described thus far produce cuboctahedral magnetite crystals that are not elongated (Amann et al., 2007). Scheffel et al. (2008) showed that the protein MamC and other proteins in the same operon (*mam*GFDC) are not essential for magnetosome formation but are involved in controlling crystal size and morphology in *M. gryphiswaldense*. In *M. australis, mamC* is organized in a *mam*CXZ operon, similar to *M. blakemorei*. The other proteins involved in the size and shape of magnetosomes (MamD, MamF, Mms6, and MmsF) are more closely related to those found in *M. marinus*. Therefore, the fact that *M. australis* MamC is related to cuboctahedral magnetite-producing bacteria suggests that this protein might be responsible for crystal morphology in this case. Additionally, based on the similarity of *mam*XZC gene organization between *M. australis* and *M. blakemorei*, we speculate that gene organization and/or preferential expression of *mam*CXZ could be involved in crystal elongation. MmsF has been shown to be involved in the geometry of magnetosome maturation, as the deletion of *mmsF* resulted in elongated magnetosomes in *M. magneticum* strain AMB-1 (Murat et al., 2012). However, we did not identify a close similarity between MmsF from *M. australis* strain IT-1 and other MTB that synthesize elongated octahedral crystals. The expression level of MmsF may influence crystal morphology, which could explain how closely related *mam* genes from different species (i.e., *M. australis* and *M. marinus*) produce magnetosomes with different characteristics. Variation in the expression level of the *mam*GFDC operon in *M. gryphiswaldense* resulted in crystals exceeding the size of those of the wild-type (Scheffel et al., 2008). The absence of *mamT* in *M. australis* strain IT-1 reveals a new group of 19 genes common to cultivated magnetotactic *Alphaproteobacteria*: *mamA, B, C, D, E, F, H, I, K, L, M, N, O, P, Q, R, S, X* and *Z*, in addition to the *mms6* and *mmsF* genes. Although *mamT* is present in the *Alpha*- and *Deltaproteobacteria*, it is not essential for biomineralization. Proteins with similar function (MamP or MamE) are likely sufficient to control the balance between Fe^2+^ and Fe^3+^ in the magnetosome. In *M. magneticum* (Murat et al., 2010) and *M. gryphiswaldense* (Lohβe et al., 2011) *mamT* is not essential for magnetosome synthesis.

Considering that both *M. australis* strain IT-1 and *M. marinus* strain MC-1 have a common magnetotactic ancestor and that biomineralization proteins apparently evolved together in both strains, it is reasonable to assume that a common ancestor exists among all freshwater and marine MTB from the *Magnetococcales* order. No non-MTB belonging to the *Magnetococcales* order has ever been reported, but this fact does not preclude HGT among *Alphaproteobacteria* because strains phylogenetically closer to *Magnetospirillum* do not have the magnetotactic phenotype (Jogler and Schüler, 2009). Thus, magnetosome biomineralization genes common to all MTB (*mamABEIKMPQ*) might have been acquired from an ancestor common to all MTB (Abreu et al., 2011; Lefèvre et al., 2013a). However, genes such as *mamCDF, mamL, mamXZ, mms6*, and *mmsF* could have been acquired by descent of magnetotactic *Alphaproteobacteria* and magnetotactic cocci, which appear to emerge as the most basal lineage of the *Alpha*- and *Gammaproteobacteria* (Singer et al., 2011; Lefèvre and Bazylinski, 2013). *mamG, mamR, mamV, mamU, and mamY* genes were likely acquired recently by *Magnetospirillum* species, given that the magnetotactic cocci studied so far, *M. marinus* strain MC-1 and *M. australis* strain IT-1, do not contain these genes. Differences observed in the biomineralization genes between *M. australis* strain IT-1, *M. marinus* strain MC-1 and the other *Alphaproteobacteria* are possibly a result of gene rearrangements, deletions or insertions of new genes through the evolution or a post-acquisition of the biomineralization genotype among MTB. Culture and sequencing of new species of magnetotactic cocci from freshwater or marine water are needed to improve the understanding the evolutionary events that occurred in the *Alphaproteobacteria* and magnetotactic cocci and will more precisely define the *Magnetococcaceae* family in the *Magnetococcales* order as either the earliest diverging order in the *Alphaproteobacteria* class or as a new class of *Proteobacteria*, as proposed by Singer et al. (2011). *M. australis* strain IT-1 is now the third cultivated magnetotactic coccus that represents a second new genus in the *Magnetococcaceae* family and is the first cultivated SS-MTB.

## Author contributions

All authors contributed to the analysis of data and composition of the paper; Viviana Morillo, Fernanda Abreu and Ana C. Araujo: experimental data acquisition and cultivation; Luiz G. P. de Almeida and Ana T. R. de Vasconcelos: pyrosequecing and bioinformatics; Alex Enrich-Prast: microeletrode measurements and interpretation; MF: high-resolution transmission electron microscopy, Viviana Morillo, Fernanda Abreu, Dennis A. Bazylinski and Ulysses Lins: analyzed data and wrote the paper.

### Conflict of interest statement

The authors declare that the research was conducted in the absence of any commercial or financial relationships that could be construed as a potential conflict of interest.
